# Taf14 is required for the stabilization of transcription pre-initiation complex in *Saccharomyces cerevisiae*

**DOI:** 10.1186/s13072-020-00347-7

**Published:** 2020-05-27

**Authors:** Kadri Peil, Henel Jürgens, Johanna Luige, Kersti Kristjuhan, Arnold Kristjuhan

**Affiliations:** 1 Institute of Molecular and Cell Biology, University of Tartu, Riia 23, 51010 Tartu, Estonia; 2 Present Address: Department of Molecular Biology and Genetics, Aarhus University, Aarhus, Denmark

**Keywords:** YEATS, Taf14, Pre-initiation complex (PIC), RNA polymerase II, Transcription, TFIIF, TFIID

## Abstract

**Background:**

The YEATS domain is a highly conserved protein structure that interacts with acetylated and crotonylated lysine residues in N-terminal tails of histones. The budding yeast genome encodes three YEATS domain proteins (Taf14, Yaf9, and Sas5) that are all the subunits of different complexes involved in histone acetylation, gene transcription, and chromatin remodeling. As the strains deficient in all these three genes are inviable, it has been proposed that the YEATS domain is essential in yeast. In this study we investigate in more detail the requirement of YEATS domain proteins for yeast survival and the possible roles of Taf14 YEATS domain in the regulation of gene transcription.

**Results:**

We found that YEATS domains are not essential for the survival of *Saccharomyces cerevisiae* cells. Although the full deletion of all YEATS proteins is lethal in yeast, we show that the viability of cells can be restored by the expression of the YEATS-less version of Taf14 protein. We also explore the in vivo functions of Taf14 protein and show that the primary role of its YEATS domain is to stabilize the transcription pre-initiation complex (PIC). Our results indicate that Taf14-mediated interactions become crucial for PIC formation in *rpb9Δ* cells, where the recruitment of TFIIF to the PIC is hampered. Although H3 K9 residue has been identified as the interaction site of the Taf14 YEATS domain in vitro, we found that it is not the only interaction target in vivo.

**Conclusions:**

Lethality of YEATS-deficient cells can be rescued by the expression of truncated Taf14 protein lacking the entire YEATS domain, indicating that the YEATS domains are not required for cell survival. The YEATS domain of Taf14 participates in PIC stabilization and acetylated/crotonylated H3K9 is not the critical target of the Taf14 YEATS domain in vivo.

## Background

The YEATS domain is a highly conserved protein domain present in more than 100 proteins from yeast to human [1]. Together with bromodomain, the double PHD finger (DPF), and the double pleckstrin homology (PH) domain proteins, it belongs to the family of the acetyllysine readers [2–5]. There are three YEATS domain proteins in budding yeast *Saccharomyces cerevisiae*: Taf14, Yaf9, and Sas5. All of them are subunits of different complexes involved in histone acetylation, gene transcription, and chromatin remodeling. Disruption of all YEATS-containing proteins is lethal in yeast, while single deletions of these genes cause relatively mild phenotypes [1, 6–9]. Although the YEATS domain was identified for more than a decade ago, several in vitro studies proposing its targets have been published just recently. It has been shown that Yaf9, the subunit of NuA4 and SWR1 complexes, binds primarily to acetylated histone H3 with a high preference for H3K27ac [10]. Another study showed that the human YEATS domain-containing protein Gas41 as well as yeast Yaf9 displayed strong binding affinity toward the succinylated H3K14, H3K56, H3K79, and H3K122 peptides in vitro, while Yaf9 did also bind the succinylated H4K12 and H4K31 [11]. Taf14, the subunit of TFIIF, TFIID, INO80, SWI/SNF, and NuA3 complexes, was first shown to have the strongest interaction with H3K9ac peptides [12], but subsequent studies specified crotonylated H3K9 (H3K9cr) as its preferred binding target [13]. Structural analysis of the Taf14 YEATS domain revealed that Phe62 and Trp81 residues of the protein form an aromatic cage, which is required for Taf14 binding to the acetylated H3K9. Mutation of Trp81 to alanine in the YEATS domain is sufficient to abolish this interaction completely [12].

The function of the YEATS domain has been just as enigmatic as its targets. As the YEATS proteins are subunits of various chromatin-modifying, or transcription-regulating complexes, it has been proposed that YEATS domains target these complexes to designated chromatin regions. For example, inactivating mutation of the Yaf9 YEATS domain was shown to impair the function of the SWR1 complex, leading to decreased deposition of H2A.Z into the *PHO5* promoter region [10]. Cells lacking Taf14 protein display reduced growth rate, sensitivity to DNA damage, and elevated temperatures, although the YEATS mutants of Taf14 cause only a slight increase of sensitivity to DNA damaging agents [12]. Furthermore, *taf14Δ* phenotype can be rescued by expression of the Taf14 C-terminal part that lacks the entire YEATS domain [14].

In this study, we tested the significance of YEATS domains for *S. cerevisiae* viability. We show that the yeast strain lacking all YEATS domain proteins, but expressing the C-terminus of Taf14, has only mild growth defect compared to wt cells. Further investigation of the Taf14 YEATS domain functions revealed that it is needed for the stabilization of the transcription pre-initiation complex (PIC) formation on gene promoters.

## Results

### Taf14 C-terminus rescues lethality of YEATS-negative cells

**Fig. 1:**
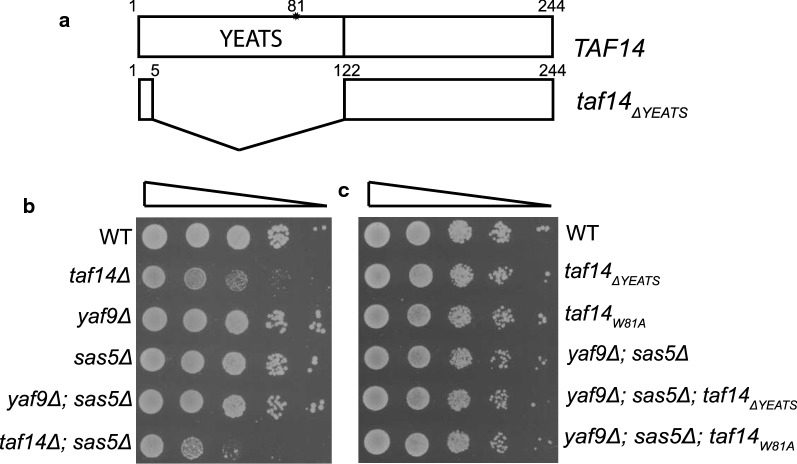
Highly conserved YEATS domain is non-essential for viability in yeast. **a** Schematic of wt and mutant Taf14 proteins used in this study. Wt Taf14 protein contains a highly conserved YEATS domain in its N-terminus. Mutant Taf14 protein without the YEATS domain (Taf14_ΔYEATS_) lacks amino acids 6–121 and mutant Taf14 protein with a non-functional YEATS domain (taf14_W81A_) has substitution of Trp81 to alanine. **b** Tenfold serial dilutions of cells with single or double-knockout of *TAF14*, *YAF9* and *SAS5* were spotted onto SC plates and grown at 30 °C for 2 days. **c** Tenfold serial dilutions of *taf14*_*ΔYEATS*_ or *taf14*_*W81A*_ cells combined with *YAF9* and *SAS5* deletions. Cells were spotted onto SC plates and grown at 30 °C for 2 days

To explore the functions of YEATS domain-containing proteins in cells, we deleted *SAS5*, *YAF9*, and *TAF14* genes in all combinations in yeast. Of single gene deletions, only *taf14Δ* cells displayed reduced growth phenotype in standard conditions (Fig. 1b). Additional deletion of *SAS5* had no synthetic effect in combination with deletions of *YAF9*, or *TAF14* (Fig. 1b), while double-knockout of *YAF9* and *TAF14* was lethal (Additional file 1: Fig. S1A). Previous studies have revealed that typical *taf14Δ* phenotype (reduced growth rate, sensitivity to DNA damaging agents, and elevated temperatures) can be rescued by expression of truncated Taf14 protein that lacks its YEATS domain [14]. Based on these observations, we hypothesized that the cause of lethality for mutant yeast strain lacking both *TAF14* and *YAF9* was not the absence of the YEATS domain as such, but rather the inability of *taf14Δ* strain to cope with extra stress resulting from deletion of *YAF9*. To test this, we replaced full-length *TAF14* in its genomic locus with genes encoding YEATS-deleted (*taf14*_*ΔYEATS*_), or YEATS-mutated (*taf14*_*W81A*_) versions of Taf14 (Fig. 1a) and combined them with deletions of *YAF9* and *SAS5* genes. As expected, *taf14*_*ΔYEATS*_ was viable in all combinations with *YAF9* and *SAS5* deletions (Additional file 1: Fig. S2), moreover, both *taf14*_*ΔYEATS*_ and *taf14*_*W81A*_ were able to rescue the strain in which all YEATS-containing proteins were deleted (Fig. 1c). This shows clearly that the lethality of *yaf9Δtaf14Δ* strain was not caused due to the lack of the YEATS domains, but rather due to the absence of the C-terminal part of Taf14.

### Synthetic phenotype of RNAPII and Taf14 YEATS mutants

**Fig. 2:**
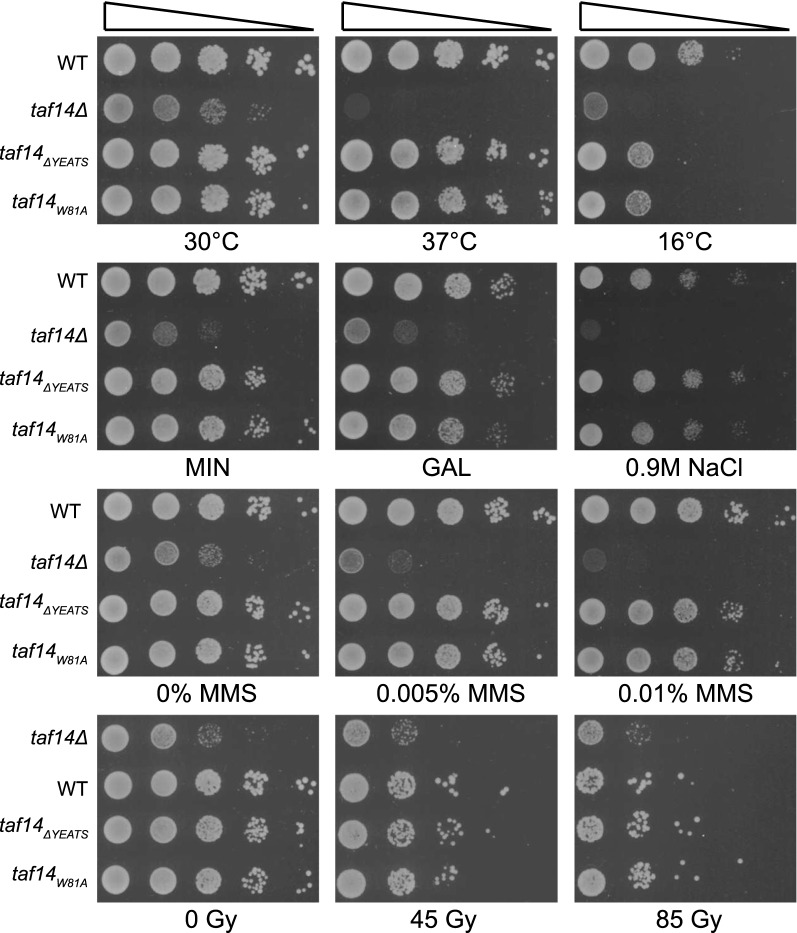
The lack of functional Taf14 YEATS domain does not cause any substantial growth defects in different growth conditions in yeast. Tenfold serial dilutions of *taf14*_*ΔYEATS*_ or *taf14*_*W81A*_ strains were spotted onto SC plates and grown at different temperatures for either 2 days (at 30 °C and 37 °C) or 5 days (at 16 °C). Cells spotted onto minimal medium (MIN) plate and cells spotted onto SC plate with galactose as a different carbon source were grown at 30 °C for 2 days. Cells spotted onto SC plate containing 0.9 M NaCl for osmotic stress were grown at 30 °C for 3 days. For DNA damage response analysis cells were spotted onto SC plates containing indicated concentrations of MMS or treatment with ionizing radiation (45–85 Gy) on SC plates was used and cells were grown at 30 °C for 2 days

To explore the functions of the Taf14 YEATS domain in more detail, we tested whether the YEATS mutants can tolerate various stress conditions (lower and higher temperature, different carbon source, osmotic stress, DNA damage). *taf14Δ* strain was sensitive to all conditions tested, while *taf14*_*ΔYEATS*_ and *taf14*_*W81A*_ cells responded to the majority of stresses in the same way as wt strain. The only exception was growth at 16 °C, where YEATS mutants displayed intermediate phenotype between wt and *taf14Δ* strains (Fig. 2). However, in contrast to our results, it has been reported that the *taf14*_*W81A*_ cells were temperature and MMS sensitive in the BY4741 strain background [12]. The discrepancy between our and previous results suggests that the requirement of the Taf14 YEATS domain might depend on strain background, or assay conditions. In previous studies, the plasmid-based expression system of Taf14 mutants was used, while our assays were performed with genomic replacement of *TAF14* with *taf14*_*W81A*_ in its native locus.

**Fig. 3:**
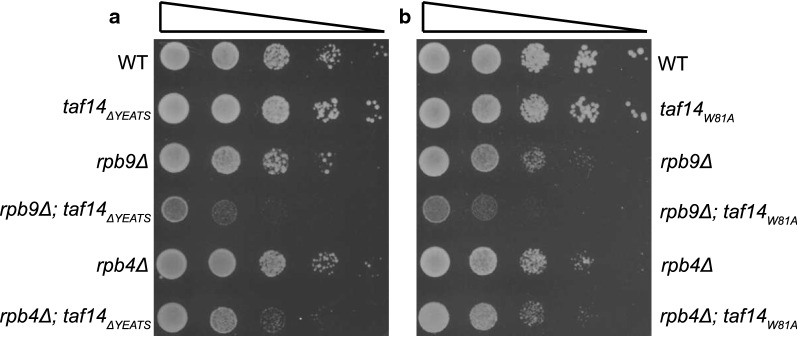
Taf14 YEATS domain is important in yeast strains with mutant RNAPII. **a** Tenfold serial dilutions *taf14*_*ΔYEATS*_ strain combined with either *RPB4* or *RPB9* deletion were spotted onto SC plates and grown at 30 °C for 2 days. **b** Tenfold serial dilutions of *taf14*_*W81A*_ strain combined with either *RPB4* or *RPB9* deletion were spotted onto SC plates and grown at 30 °C for 2 days

As all Taf14-containing protein complexes are involved directly or indirectly in gene transcription, we tested whether Taf14 YEATS mutants may have more evident effects on regulation of RNAPII-dependent transcription. To make cells more vulnerable for minor disturbances in transcription, we used *rpb4Δ* and *rpb9Δ* cells to test Taf14 YEATS mutants. In yeast, Rpb4 and Rpb9 are non-essential subunits of RNAPII, although in the absence of either protein cells grow slower and display several defects in transcription initiation [15–19]. We found that the deletion of *TAF14* was lethal in *rpb4Δ* and *rpb9Δ* background, while Taf14 YEATS mutants were viable (Fig. 3 and Additional file 1: Figs. S1B–E). However, the YEATS mutants had synthetic phenotype with *rpb4Δ* and *rpb9Δ*, underlining the substantial role of the Taf14 YEATS domain in these strains (Fig. 3).

### H3K9 is not the only target of the Taf14 YEATS domain in vivo

**Fig. 4:**
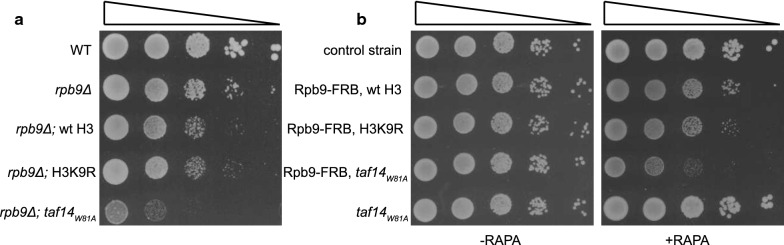
Modified H3K9 is not the only target of the Taf14 YEATS domain. **a** Tenfold serial dilutions of *rpb9Δ* cells expressing either wt H3 or mutant H3K9R histones and *rpb9Δ* cells expressing *taf14*_*W81A*_ were spotted onto SC plates and grown at 30 °C for 2 days. **b** Tenfold serial dilutions of Rpb9 anchor-away cells expressing either wt H3 or mutant H3K9R histones and Rpb9 anchor-away cells expressing *taf14*_*W81A*_ were spotted onto SC plates in the absence (−RAPA) or presence of rapamycin (+RAPA) and grown at 30 °C for 2 days. Strain without a functional anchor-away system (AKY1159) was used as a control

Recent studies have indicated that the Taf14 YEATS domain interacts specifically with acetylated and crotonylated N-terminal tails of H3 [12, 13]. Although modification of several different lysine residues in H3 N-terminal peptides supported the recruitment of Taf14 YEATS domain, and multiple modifications on the same peptide had a cumulative effect in these assays, the acetylated/crotonylated H3K9 was identified as the primary target of Taf14 YEATS domain in vitro [12, 13, 20]. The synthetic phenotype of Taf14 YEATS mutants with *rpb4Δ* and *rpb9Δ* strains suggests that recognition of modified H3K9 by Taf14 may become more critical in the absence of these proteins. To test whether H3K9 is the primary target of Taf14 also in vivo, we compared the growth rates of *rpb9Δ* strains carrying either *taf14*_*W81A*_, or K9R mutation in histone H3, which does not allow its modification. If acetylated or crotonylated H3K9 is the primary target of Taf14 in vivo, the *H3K9R* strain should express the same level of genetic interaction with *rpb9Δ* as does the *taf14*_*W81A*_ strain. While *taf14*_*W81A*_ displayed a clear synthetic phenotype with *rpb9Δ*, the *H3K9R* mutation did not distinguish from *rpb9Δ* strain carrying wt H3 (Fig. 4a). An identical result was obtained when Rpb9 was removed from the nucleus by the anchor-away technique (Fig. 4b). The latter approach allows to pre-grow cells in wt conditions and remove Rpb9 just before the assay, thus avoiding possible adaptation of the strain for growth in the absence of Rpb9. These results show that acetylated/crotonylated H3K9 is not the critical target of Taf14 YEATS domain in vivo, suggesting that other modified lysine residues in histone tails, or in non-histone proteins can compensate the lack of H3K9, providing the alternative docking sites for Taf14 binding. Our previous study showed that none of the single lysine residue mutations in the H3 N-terminal tail had synthetic phenotype with *RPB9* deletion [21], supporting the idea that H3 tail modifications are functionally redundant. Unfortunately, we cannot test combined mutations of multiple H3 N-terminal lysines in the *rpb9Δ* background, as this leads to genomic instability and inviability of the cells due to inefficient activation of DNA damage checkpoint response pathway [21].

### Taf14 YEATS is necessary for the stabilization of the pre-initiation complex

**Fig. 5:**
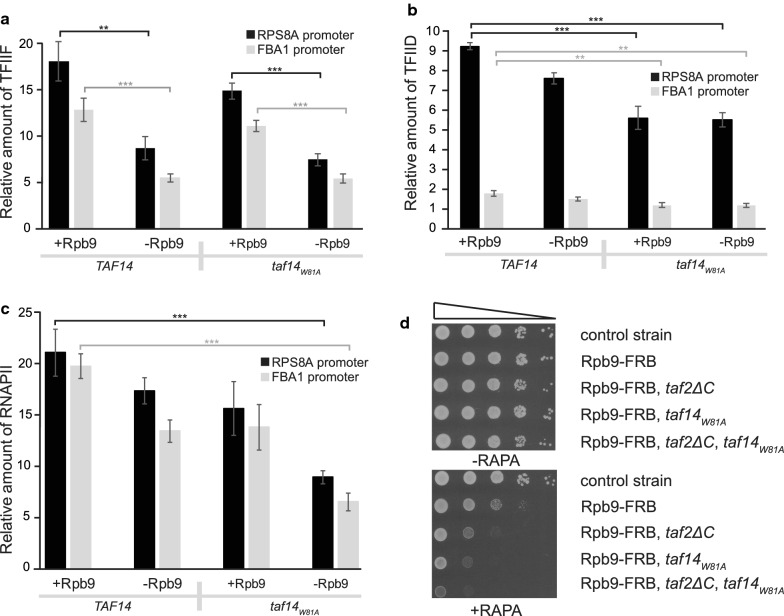
The simultaneous absence of Rpb9 and inactivation of Taf14 YEATS has a cumulative effect in the reduction of the relative levels of RNAPII on promoters. The relative amount of TFIIF (**a**), TFIID (**b**), and RNAPII (**c**) at the highly expressed *FBA1* and *RPS8A* gene promoters in the indicated strains was analyzed with ChIP and qPCR. Rpb9 was removed from the cell nucleus by the anchor-away technique in the presence of rapamycin. Tfg2 (TFIIF) and Taf2 (TFIID) were C-terminally tagged with FLAG tag, Rpb3 (RNAPII) was C-terminally tagged with E2-tag. Non-coding region in the right arm of ChrVI telomere was used as an internal control. Error bars represent the standard deviation of at least four independent experiments. **d** Tenfold serial dilutions of indicated anchor-away cells were spotted onto SC plates in the absence or presence of rapamycin and grown at 30 °C for 2 days. ** indicates *p* ≤ 0.01 and *** indicates *p* ≤ 0.001

Taf14 is a subunit of two basal transcription factors, TFIID and TFIIF that are both required for PIC formation. Considering that Taf14 YEATS mutants were not distinguishable from wt cells upon exposure to genotoxic stress (Fig. 2), but had synthetic phenotype with RNAPII subunits (Fig. 3), we decided to test whether Taf14 YEATS mutants affect the efficiency of PIC assembly. We used Rpb9 anchor-away strain carrying either wt *TAF14* or *taf14*_*W81A*_ and measured the relative amounts of RNAPII, TFIIF, and TFIID complexes on promoters of two highly expressed genes *FBA1* and *RPS8A*. Among the PIC components, TFIIF is the most obvious common target of Taf14 and Rpb9. Taf14 is a subunit of TFIIF and on the other hand, in vitro interaction between purified RNAPII and TFIIF complexes is strongly reduced in the absence of Rpb9 [22]. Although there was a slight reduction of TFIIF on promoters in *taf14*_*W81A*_ cells compared to their wt counterparts, exclusion of Rpb9 from cell nucleus had a more significant effect on the recruitment of TFIIF, regardless of the status of Taf14 (Fig. 5a). This confirms that Rpb9 is the major interaction partner of TFIIF, although some of TFIIF is recruited to the PIC independently of Rpb9. On the other hand, the presence of TFIID on gene promoters was reduced in *taf14*_*W81A*_ cells, while removal of Rpb9 had only minor effect for TFIID occupancy. Notably, the reduction of TFIID level was dependent mainly on *taf14*_*W81A*_ mutation, as depletion of Rpb9 did not lead to further loss of TFIID in *taf14*_*W81A*_ cells (Fig. 5b). This suggests that the most likely role of Taf14 is to stabilize PIC components on gene promoters via YEATS-mediated interactions. We observed a relatively low amount of TFIID on *FBA1* promoter in all conditions, which is in accordance with previous findings that the *RPS8A* promoter is strongly dependent on TFIID, while the *FBA1* promoter is not [23, 24]. A relatively small reduction of TFIID occupancy in *taf14*_*W81A*_ cells is in concordance with the mild phenotype of the Taf14 YEATS mutant strains and indicates supportive, but not critical role of Taf14 in TFIID stabilization. In contrast, the occupation of RNAPII was reduced by depletion of Rpb9 and inactivation of the Taf14 YEATS domain, and a combination of both factors had a cumulative effect in the reduction of RNAPII on promoters (Fig. 5c). Collectively, these results suggest that cells can tolerate moderate instability of either TFIID or TFIIF on promoters, but a simultaneous weakening of both interactions results in substantial loss of RNAPII recruitment to the PIC, which in turn is reflected in the synthetic phenotype of *rpb9Δ taf14*_*W81A*_ cells.

Although these results suggest that Taf14 is required primarily for TFIID stabilization, it has to be considered that Taf14 is also a subunit of TFIIF, and therefore, *taf14*_*W81A*_ mutation affects both of these complexes. To evaluate whether the synthetic phenotype of *taf14*_*W81A*_ in the absence of Rpb9 was primarily caused by the impediment of TFIID, or TFIIF, we tested the effect of Rpb9 depletion in the *taf2ΔC* strain background. In this strain, the last 147 amino acids from Taf2 C-terminus are deleted, which disrupts Taf14 interaction with TFIID [25]. In *taf2ΔC* cells, fully functional Taf14 is expressed and incorporated into all Taf14-containing complexes, except TFIID. When Rpb9 was depleted in this stain background, the growth of cells was similar to the Rpb9-depleted *taf14*_*W81A*_ cells, suggesting that Taf14 interaction with TFIID becomes critical for cell growth in the absence of Rpb9 (Fig. 5d). However, we cannot rule out that the deletion of Taf2 C-terminus does not affect other functions, or interactions of TFIID that may lead to the synthetic phenotype with Rpb9 depletion independently from TFIID–Taf14 interaction. In fact, when *taf14*_*W81A*_ was combined with the deletion of the C-terminus of Taf2, the synthetic phenotype with Rpb9 depletion was even stronger than by either mutation alone (Fig. 5d).

## Discussion

It has been proposed that the YEATS domain is essential for cells, as the deletion of all three YEATS-containing proteins is lethal in yeast [1, 9]. This assumption would imply that in the absence of one YEATS domain protein, other YEATS domain proteins could substitute its function. However, this scenario is rather unlikely, as the complexes containing either Yaf9, Sas5, or Taf14 are required for different functions in the cell. To explore the roles and requirements of YEATS proteins in vivo, we deleted all the three genes in yeast and found that the lethality of *yaf9Δsas5Δtaf14Δ* triple-knockout strain can be fully rescued by the expression of a truncated version of Taf14 protein, which lacks the entire YEATS domain. Therefore, the YEATS domain is dispensable for viability of budding yeast, if the C-terminal part of Taf14 is expressed in cells. Previous studies have demonstrated that Taf14 C-terminus is required for its incorporation into TFIID, TFIIF, SWI/SNF, INO80, and NuA3 complexes [14]. On the other hand, the integrity and enzymatic activity of these complexes were not substantially affected in the absence of Taf14, suggesting that it was not required for the basic functions of the complexes [25–30]. This observation is also supported by the fact that Taf14 is the only non-essential subunit in TFIID and TFIIF, and inactivation of catalytic subunits in SWI/SNF, or INO80 complexes leads to far more severe phenotypes than deletion of *TAF14* gene [31, 32]. Based on these observations, it has been proposed that the main role of Taf14 is to recognize histone modifications via its YEATS domain and target the protein complexes to the designated chromatin regions. However, as the YEATS-less Taf14 can compensate for the lack of all YEATS-containing proteins, it suggests that YEATS-independent roles of Taf14 may have been underestimated and the functions of Taf14 in protein complexes are not restricted to its YEATS domain.

It has been shown that Taf14 YEATS interacts with acetylated and crotonylated histone H3 N-terminal peptides [12, 13]. Modifications of H3K9 residue have been identified as the primary targets of Taf14 in vitro, although these studies also revealed that Taf14 interaction with polyacetylated H3 peptides was even better than with mono-acetylated H3K9ac peptide [33]. However, the specificity of Taf14 interactions has not been confirmed in vivo. The main obstacle for testing the Taf14 target sites in vivo is that the strains harboring Taf14 YEATS mutations display only very mild phenotypes [12, 14]. To enhance the influence of Taf14 YEATS domain mutations in vivo, we were searching for the synthetic phenotype of Taf14 YEATS mutants with other transcription factors and found that the strains lacking either Rpb4 or Rpb9 subunits of RNAPII displayed severe growth defect in *taf14*_*ΔYEATS*_ and *taf14*_*W81A*_ background. This opened a unique opportunity to test the possible targets of the Taf14 YEATS domain in vivo. We assumed that if H3K9 is the only target of Taf14, then elimination of any possible modification of H3K9 in *rpb9Δ* cells should copy the phenotype of Taf14 YEATS mutants in the *rpb9Δ* background. Surprisingly, we found no synthetic phenotype of *H3K9R* mutation with *rpb9Δ*, indicating that acetylated/crotonylated H3K9 cannot be the only binding target of Taf14 in vivo. This suggests that the Taf14 YEATS domain can fulfill its functions also through alternative interaction sites in chromatin, if H3K9 modifications are not available. It is also possible that lysine modifications of non-histone proteins may provide alternative binding sites for the Taf14 YEATS domain in vivo.

Although the Taf14 protein is present in many chromatin- and transcription-related protein complexes, its role in transcription regulation has remained obscure. Considering that Taf14 YEATS mutants had synthetic phenotype with RNAPII subunits, we tested whether these mutants affect the efficiency of PIC assembly. The formation of PIC requires coordinated recruitment of approximately 60 proteins [34, 35] that are stabilized by multiple, and often redundant, interactions between its components. Elimination of some of these interactions does not necessarily cause the dramatic failure of complex formation, however, the abolition of multiple interactions may lead to cumulative effect and destabilization of the whole complex. Our results indicate that the YEATS domain of Taf14 is one of those factors that contribute to the formation and stabilization of PIC. While its effect alone is rather minor, it becomes more prominent, when the recruitment of TFIIF to the PIC is hampered. Although the levels of all tested PIC components (TFIID, TFIIF, and RNAPII) were slightly reduced in the *taf14*_*W81A*_ strain background, the strongest reduction was seen in the recruitment of TFIID. Stoichiometry of purified TFIID indicates that it contains multiple copies of Taf14 protein and also at least two Taf14 binding domains have been identified in the C-terminus of Taf2 [25, 36]. Therefore, the stability of TFIID in the PIC may be more dependent on Taf14-mediated interactions than the stability of other PIC components. Remarkably, the deletion of Taf2 C-terminus and *taf14*_*W81A*_ mutation had a similar synthetic phenotype with depletion of Rpb9, suggesting that Taf14 is primarily required as a subunit of the TFIID complex in Rpb9-deficient cells. However, we saw that *taf14*_*W81A*_*taf2ΔC* strain had an even stronger synthetic phenotype with Rpb9 depletion than either mutation alone. This suggests that the C-terminus of Taf2 may have more functions in TFIID than providing a binding domain for Taf14, and therefore it may affect PIC stability also independently from Taf14. For example, a recent study of *Komagataella phaffii* TFIID structure revealed that Taf14 binds to Taf2 side-by-side with the Taf8 subunit [37], suggesting a possible role of Taf2 C-terminal domain in orchestrating the correct arrangement of these subunits in TFIID. Also, it has to be considered that the deletion of the Taf2 C-terminal domain fully eliminates incorporation of Taf14 to the TFIID complex, while the Taf14 YEATS domain mutants are expected to interact with all Taf14-containing complexes. Moreover, as the expression of the YEATS-less Taf14 can rescue the phenotypes of *taf14Δ* cells, the YEATS-independent functions of Taf14 may be as important as its interactions through the YEATS domain. At least two possible mechanisms can explain this phenomenon. First, although the lack of Taf14 from different protein complexes seems not to affect the activities of these complexes, it may stabilize the active conformation of these complexes. For example, the interaction of Taf14 with Taf2 can compensate some of the *taf2*-*ts* mutants in vivo, although these mutations are located outside of the Taf14-binding sites on Taf2 [25]. Second, Taf14 can form dimers in vitro [38], suggesting that it may facilitate inter-complex interactions via Taf14–Taf14 dimerization. In this respect, the C-terminal domain of Taf2 might serve as “a landing platform” for the various Taf14-containing complexes during the PIC formation.

Neither Taf14 YEATS mutations, nor the deletion of Taf2 C-terminus causes any remarkable phenotype when present alone, or combined. However, all these mutations have synthetic phenotype with inactivation of Rpb9. The most obvious common target of these mutations is the TFIIF complex, which contains Taf14 as its subunit, and its interaction with RNAPII is strongly dependent on Rpb9 [22]. Therefore, it is likely that the diminished recruitment, or stabilization of TFIIF into the PIC is the major reason for the synthetic phenotype. First of all, in the absence of Rpb9, the interaction between TFIIF and RNAPII is weak and the stable binding of both factors to the PIC becomes more dependent on other protein–protein interactions. We propose that in this situation the Taf14-mediated interactions become critical for efficient PIC formation. Taf14 can stabilize the PIC by providing TFIID and TFIIF additional modules for interaction with chromatin (via the YEATS domains) or with each other (via Taf14–Taf14 dimerization). When one of these supporting interactions is disabled in Rpb9-deficient cells either due to the inactivation of the Taf14 YEATS domain, or by the deletion of the Taf2 C-terminus, the synthetic phenotype appears. When both these interactions are abolished in the *taf14*_*W81A*_*taf2ΔC* strain, the synthetic phenotype becomes even more severe than by either mutation alone.

Interestingly, all the major components of this network are found in yeasts, but not in higher eukaryotes. In metazoans, the Taf2 proteins do not contain the C-terminal domain that is found in yeasts and concordantly, the TFIID complex does not contain the Taf14 subunit. Also, the metazoan TFIIF contains two subunits, lacking the third, Taf14, which is present in yeast TFIIF. In addition, deletion of Rpb9 is lethal in higher eukaryotes, while yeasts can survive without this subunit of RNAPII. Collectively, these observations suggest that yeasts may have a Taf14-based back-up system for recruitment of TFIIF to gene promoters, while metazoans rely mostly on direct interactions between TFIIF and RNAPII. The benefits of this back-up system are not clear, although it might allow faster and more flexible responses of unicellular organisms to the changes in their growth environment, for example in response to the availability of nutrients, or fluctuations of temperature.

## Conclusions

In this study, we show that the highly conserved YEATS domain is not required for cell viability in budding yeast. Although the Taf14 YEATS domain interacts specifically with acetylated and crotonylated histone H3 K9 in vitro [12, 13], our data indicate that it is not the critical target of the Taf14 YEATS domain in vivo. Furthermore, we show that Taf14 YEATS domain supports the formation of PIC on gene promoters by stabilizing TFIID and TFIIF binding to the complex.

## Materials and methods

### Yeast strains, plasmids, and antibodies

All *Saccharomyces cerevisiae* strains were derived from the W303 background [39] and are listed in Additional file 1: Table S1. Strains AKY1027 (*rpb9Δ*) and 1158 (Rpb9 anchor-away) were used in plasmid shuffling assays. These strains express wild type copies of *HHT2* and *HHF2* from a *URA3*-based plasmid (YCp50:HHT2-HHF2) as a sole source for histones H3 and H4. Histone H3K9R point mutation was made in *HIS3*-based plasmid (pRS413-H3H4-3F12). Either wt or H3K9R plasmid was transformed into AKY1027 or AKY1158, and counter-selected on 5-fluoroorotic acid (5-FOA) plates (1 mg/ml) to obtain strain with a wt or a mutant H3. Rpb9 anchor-away strains were derived from strain HHY168 (Euroscarf) [40], where *RPB9* locus was replaced with *rpb9*-*FRB*-*hphMX* expression cassette. In the presence of rapamycin Rpb9-FRB is depleted from the nucleus by conditional tethering to the “anchor” Rpl13a protein, containing C-terminal FKBP12-tag. In control strain (AKY1159) Rpb9 protein is still C-terminally tagged with FRB-tag, but *RPL13A* locus lacks the FKBP12-tag. We confirmed that in these strains both wt Taf14 and Taf14_W81A_ proteins were expressed in equal levels before and after the removal of Rpb9 from the cell nucleus (Additional file 1: Fig. S3). Yeast strains expressing Taf14_ΔYEATS_ (lacking amino acids 6-121) and Taf14_W81A_ were generated by replacement of genomic *TAF14* locus with either *spHIS5*-*taf14*_*ΔYEATS*_ or *spHIS5*-*taf14*_*W81A*_ expression cassette. The intron sequence of Taf14 was present in these expression cassettes. RNAPII Rpb3 subunit was C-terminally tagged with E2-tag and detected with 5E11 antibody (Icosagen), Tfg2, and Taf2 were C-terminally tagged with FLAG tag and detected with M2 antibody (Sigma-Aldrich). Taf14 was detected with an anti-Taf14 antibody (A278, antibodies.com). For Western blot, cell extracts were prepared as described [41] and protein samples were separated on SDS–polyacrylamide gel.

### Yeast growth assays

Culture density was measured with Z2 Cell and Particle Counter (Beckman Coulter). For spot test assays, tenfold serial dilutions of cell suspensions were made and 5 µl of each dilution was spotted onto plates with synthetic complete (SC) selective medium. Indicated concentrations of methyl methanesulfonate (MMS) in SC plates were used to test the viability of cells. Cells were also treated with ionizing radiation (45–85 Gy). In experiments with Rpb9 anchor-away strains, 1 µg/ml rapamycin (Cayman Europe) in 0.1% DMSO as a final concentration was added to the cultures (0.1% DMSO was used for controls). Plates were incubated for 2 days at 30 °C, unless otherwise stated.

### Chromatin immunoprecipitation (ChIP) assay

Yeast cultures were inoculated into 25 ml fresh YPD media at density 8 × 10^6^ cells per ml. After incubation 120 min in a shaker at 30 °C, either DMSO (0.1% final concentration) or rapamycin (1 µg/ml) in 0.1% DMSO was added and cells were cultured for another 120 min. Cells were fixed in 1% formaldehyde for ChIP assay. ChIP assays were performed as described previously [42]. Whole-cell extract from 1 × 10^7^ cells was used for ChIP assays with antibodies directed against anti-E2 tag or anti-FLAG tag. Co-precipitated DNA was analyzed by quantitative real-time PCR using LightCycler 480 Real-Time PCR System under standard conditions (40 cycles, 95 °C for 15 s + 60 °C for 1 min). Maxima SYBR Green/ROX qPCR master mix (Thermo Scientific) was used. PCRs were performed with primer pairs covering the promoter regions of *FBA1* and *RPS8A*. Non-transcribed region in chromosome VI right arm telomere was used as an internal control and for normalization of ChIP results. The primer sequences used in these analyses are as follows: FBA1algF 5′GAGAAAGACCGGTGTCATCGTTGG3′; FBA1algR 5′CCTTACCAGCGAAGTAAGCAGCAC3′; RPS8ApromF 5′CAGGACCTCTCTTTGAATGGAATAG3′; RPS8ApromR 5′CTTCTTGTGAAAAACTCGGCGTTTC3′; Tel6RF 5′TAACAAGCGGCTGGACTACTTTC3′; Tel6RR 5′GATAACTCTGAACTGTGCATCCACTC3′. Data were obtained from at least four different experiments. Error bars represent the standard deviation between the biological replicates. Student *t* test was used when comparing mean differences of two experimental groups. The level of statistical significance was established at a *p* value of < 0.05, ** indicates *p* ≤ 0.01, and *** indicates *p* ≤ 0.001.

## Supplementary information

**Additional file 1: Figure S1**. Deletion of TAF14 is lethal in yaf9Δ, rpb4Δ and rpb9Δ cells in W303 background, but functional C-terminal domain of Taf14 rescues rpb4Δtaf14Δ and rpb9Δtaf14Δ lethality. Tetrad analysis following sporulation of AKY1820+AKY1916 (A), AKY1786+AKY1818 (B), AKY718+AKY1818 (C), AKY1850+1938 (D) and AKY719+AKY1850 (E) yeast strains. The tetrads were dissected on YPD medium and plates photographed after 4 days of growth at 30 °C. **Figure S2.** Expression of C-terminal domain of Taf14 rescues yaf9Δtaf14Δ double-mutant from lethality. Tenfold serial dilutions of cells, where TAF14 in its genomic locus is replaced with gene encoding YEATS-deleted Taf14 protein (taf14ΔYEATS) and combined with YAF9 and SAS5 deletions, were spotted onto SC plates and grown at 30 °C for 2 days. **Figure S3.** Western blot analysis of Taf14 (A) and Rpb3 (B) in response to Rpb9 depletion. Rpb9 anchor-away strains with wt Taf14 or mutant taf14W81A were incubated with DMSO (+Rpb9) or rapamycin (−Rpb9) for 2 h. Taf14 was detected with anti-Taf14 antibody, RNAPII Rpb3 subunit was C-terminally tagged with E2-tag and detected with 5E11 antibody. A sample from taf14Δ strain expressing Rpb3 without E2-tag was used as a negative control (N). **Table S1.** Yeast strains.

## Data Availability

All data generated or analyzed during this study are included in this published article and its additional files.

## Abbreviations

PIC: Pre-initiation complex
RNAPII: RNA polymerase II
TFIID: Transcription factor IID
TFIIF: Transcription factor IIF
