# Supplementary material for: Taf14 is required for the stabilization of transcription pre-initiation complex in Saccharomyces cerevisiae
Source: Epigenetics Chromatin. 2020 May 27;13:24. doi: 10.1186/s13072-020-00347-7 (PMC7254723; doi:10.1186/s13072-020-00347-7)
Supplement: Supplementary file 1 — Additional file 1: Figure S1. Deletion of TAF14 is lethal in yaf9Δ, rpb4Δ and rpb9Δ cells in W303 background, but functional C-terminal domain of Taf14 rescues rpb4Δtaf14Δ and rpb9Δtaf14Δ lethality. Tetrad analysis following sporulation of AKY1820+AKY1916 (A), AKY1786+AKY1818 (B), AKY718+AKY1818 (C), AKY1850+1938 (D) and AKY719+AKY1850 (E) yeast strains. The tetrads were dissected on YPD medium and plates photographed after 4 days of growth at 30 °C. Figure S2. Expression of C-terminal domain of Taf14 rescues yaf9Δtaf14Δ double-mutant from lethality. Tenfold serial dilutions of cells, where TAF14 in its genomic locus is replaced with gene encoding YEATS-deleted Taf14 protein (taf14ΔYEATS) and combined with YAF9 and SAS5 deletions, were spotted onto SC plates and grown at 30 °C for 2 days. Figure S3. Western blot analysis of Taf14 (A) and Rpb3 (B) in response to Rpb9 depletion. Rpb9 anchor-away strains with wt Taf14 or mutant taf14W81A were incubated with DMSO (+Rpb9) or rapamycin (−Rpb9) for 2 h. Taf14 was detected with anti-Taf14 antibody, RNAPII Rpb3 subunit was C-terminally tagged with E2-tag and detected with 5E11 antibody. A sample from taf14Δ strain expressing Rpb3 without E2-tag was used as a negative control (N). Table S1. Yeast strains. [file 13072_2020_347_MOESM1_ESM.pdf]

A

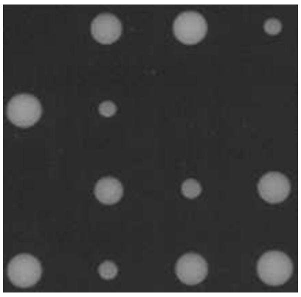

taf14<br>*yaf9*	*TAF14*<br>*yaf9*	*TAF14*<br>*YAF9*	*taf14*<br>*YAF9*
TAF14<br>*YAF9*	*taf14*<br>*YAF9*	*taf14*<br>*yaf9*	*taf14*<br>*yaf9*
taf14<br>*yaf9*	*TAF14*<br>*yaf9*	*taf14*<br>*YAF9*	*TAF14*<br>*yaf9*
TAF14<br>*YAF9*	*taf14*<br>*YAF9*	*TAF14*<br>*yaf9*	*TAF14*<br>*YAF9*

B

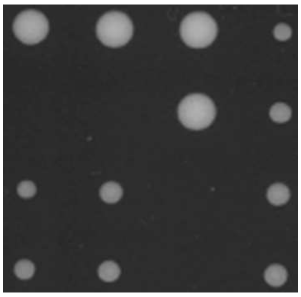

TAF14<br>*RPB4*	*TAF14*<br>*RPB4*	*TAF14*<br>*RPB4*	*taf14*<br>*RPB4*
taf14<br>*rpb4*	*taf14*<br>*rpb4*	*TAF14*<br>*RPB4*	*taf14*<br>*RPB4*
TAF14<br>*rpb4*	*TAF14*<br>*rpb4*	*taf14*<br>*rpb4*	*TAF14*<br>*rpb4*
taf14<br>*RPB4*	*taf14*<br>*RPB4*	*taf14*<br>*rpb4*	*TAF14*<br>*rpb4*

C

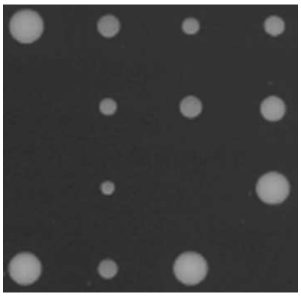

TAF14<br>*RPB9*	*TAF14*<br>*rpb9*	*taf14*<br>*RPB9*	*taf14*<br>*RPB9*
taf14<br>*rpb9*	*taf14*<br>*RPB9*	*TAF14*<br>*rpb9*	*TAF14*<br>*rpb9*
taf14<br>*rpb9*	*taf14*<br>*RPB9*	*taf14*<br>*rpb9*	*TAF14*<br>*RPB9*
TAF14<br>*RPB9*	*TAF14*<br>*rpb9*	*TAF14*<br>*RPB9*	*taf14*<br>*rpb9*

D

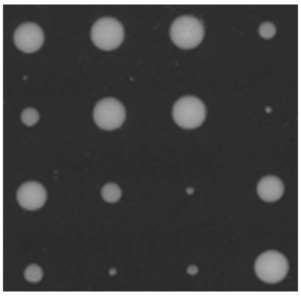

$taf14_{\Delta YEATS}$<br>*RPB4*	*TAF14*<br>*RPB4*	*TAF14*<br>*RPB4*	*TAF14*<br>*rpb4*
TAF14<br>*rpb4*	$taf14_{\Delta YEATS}$<br>*RPB4*	*TAF14*<br>*RPB4*	$taf14_{\Delta YEATS}$<br>*rpb4*
$taf14_{\Delta YEATS}$<br>*RPB4*	*TAF14*<br>*rpb4*	$taf14_{\Delta YEATS}$<br>*rpb4*	$taf14_{\Delta YEATS}$<br>*RPB4*
TAF14<br>*rpb4*	$taf14_{\Delta YEATS}$<br>*rpb4*	$taf14_{\Delta YEATS}$<br>*rpb4*	*TAF14*<br>*RPB4*

E

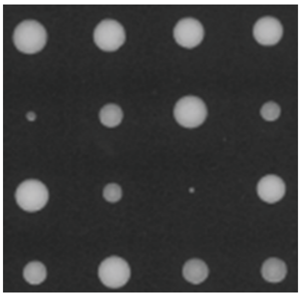

TAF14<br>*RPB9*	$taf14_{\Delta YEATS}$<br>*RPB9*	$taf14_{\Delta YEATS}$<br>*RPB9*	$taf14_{\Delta YEATS}$<br>*RPB9*
$taf14_{\Delta YEATS}$<br>*rpb9*	*TAF14*<br>*rpb9*	*TAF14*<br>*RPB9*	*TAF14*<br>*rpb9*
$taf14_{\Delta YEATS}$<br>*RPB9*	*TAF14*<br>*rpb9*	$taf14_{\Delta YEATS}$<br>*rpb9*	$taf14_{\Delta YEATS}$<br>*RPB9*
TAF14<br>*rpb9*	$taf14_{\Delta YEATS}$<br>*RPB9*	*TAF14*<br>*rpb9*	*TAF14*<br>*rpb9*

Figure S1. Deletion of TAF14 is lethal in *yaf9Δ*, *rpb4Δ* and *rpb9Δ* cells in W303 background, but functional C-terminal domain of Taf14 rescues *rpb4Δtaf14Δ* and *rpb9Δtaf14Δ* lethality. Tetrad analysis following sporulation of AKY1820+AKY1916 (**A**), AKY1786+AKY1818 (**B**), AKY718+AKY1818 (**C**), AKY1850+1938 (**D**) and AKY719+AKY1850 (**E**) yeast strains. The tetrads were dissected on YPD medium and plates photographed after 4 days of growth at 30 °C.

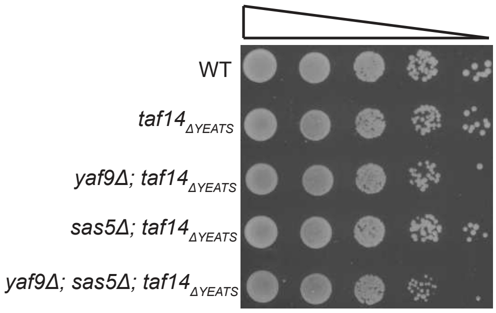

Figure S2. Expression of C-terminal domain of Taf14 rescues *yaf9Δtaf14Δ* double-mutant from lethality. Tenfold serial dilutions of cells, where TAF14 in its genomic locus is replaced with gene encoding YEATS-deleted Taf14 protein (taf14ΔYEATS) and combined with *YAF9* and *SAS5* deletions, were spotted onto SC plates and grown at 30 °C for 2 days.

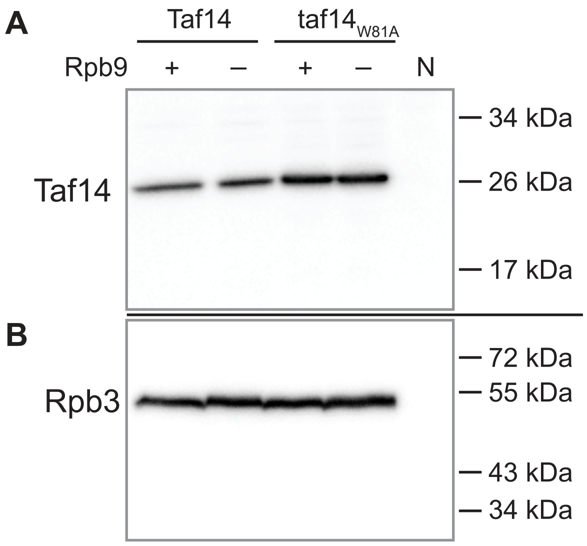

Figure S3. Western blot analysis of Taf14 (**A**) and Rpb3 (**B**) in response to Rpb9 depletion. Rpb9 anchor-away strains with wt Taf14 or mutant $taf14_{W81A}$ were incubated with DMSO (+Rpb9) or rapamycin (-Rpb9) for 2 hours. Taf14 was detected with anti-Taf14 antibody, RNAPII Rpb3 subunit was C-terminally tagged with E2-tag and detected with 5E11 antibody. A sample from *taf14Δ* strain expressing Rpb3 without E2-tag was used as a negative control (N).

Table S1. Yeast strains

Strain	Genotype	Source
AKY152	W303 *MAT A ura3-1 leu2-3,112 his3-11,15 trp1-1 ade2-101 lys2D can1-100*	[39]
AKY718	*MAT A, rpb9::natMX6*	This study
AKY1027	*MAT A, hht1-hhf1::LEU2 gal-pr-vps13::TRP1 hht2-hhf2::kanMX rpb9::natMX6 YCp50:hht2-hhf2 (URA3)*	This study
AKY1158	*MAT A, rpl13a-2-fkbp12::TRP1 fpr1::natMX6 tor1-1 rpb9-frb::hghMX hht1-hhf1::LEU2 hht2-hhf2::kanMX YCp50:hht2-hhf2 (URA3)*	This study
AKY1159	*MAT A, fpr1::natMX6 tor1-1 rpb9-frb::hghMX hht1-hhf1::LEU2 hht2-hhf2::kanMX YCp50:hht2-hhf2 (URA3)*	This study
AKY1538	*MAT A, rpl13a-2-fkbp12::TRP1 fpr1::natMX6 tor1-1 rpb9-frb::hghMX*	This study
AKY1819	*MAT A, taf14::KanMX*	This study
AKY1821	*MAT A, yaf9::TRP1*	This study
AKY1823	*MAT A, sas5:: hghMX*	This study
AKY1825	*MAT A, sas5::hghMX yaf9::TRP1*	This study
AKY1827	*MAT A, sas5::hghMX taf14::KanMX*	This study
AKY1850	*MAT A, taf14Δ6-121::spHIS5*	This study
AKY1871	*MAT A, TAF14::spHIS5*	This study
AKY1934	*MAT A, taf14Δ6-121::spHIS5 rpb9::natMX6*	This study
AKY1939	*MAT A, TAF14::spHIS5 rpb9::natMX6*	This study
AKY1963	*MAT A, TAF14::spHIS5 rpb4::LEU2*	This study
AKY1964	*MAT A, taf14Δ6-121::spHIS5 rpb4::LEU2*	This study
AKY1987	*MAT A, rpb4::LEU2*	This study
AKY2160	*MAT A, taf14W81A::spHIS5*	This study
AKY2174	*MAT A, taf14W81A::spHIS5, rpb9::natMX6*	This study
AKY2177	*MAT A, taf14Δ6-121::spHIS5 sas5::hghMX yaf9::TRP1*	This study
AKY2179	*MAT A, taf14W81A::spHIS5 rpb4::LEU2*	This study
AKY2181	*MAT A, taf14W81A::spHIS5 sas5::hghMX yaf9::TRP1*	This study
AKY2185	*MAT A, taf14W81A::spHIS5 rpl13a-2-fkbp12::TRP1 fpr1::natMX6 tor1-1 rpb9-frb::hghMX*	This study
AKY2192	*MAT A, rpl13a-2-fkbp12::TRP1 fpr1::natMX6 tor1-1 rpb9-frb::hghMX rpb3-3x3F12::LEU2 tfg2-3xFlag::URA3*	This study
AKY2193	*MAT A, taf14W81A::spHIS5 rpl13a-2-fkbp12::TRP1 fpr1::natMX6 tor1-1 rpb9-frb::hghMX rpb3-3x3F12::LEU2 tfg2-3xFlag::URA3*	This study
AKY2194	*MAT α, taf14W81A::spHIS5 fpr1::natMX6 tor1-1 rpb9-frb::hghMX hht1-hhf1::LEU2 hht2-hhf2::kanMX YCp50:hht2-hhf2 (URA3)*	This study

AKY2207	*MAT A, taf2Δ1261-1407-3xFlag::KanMX rpl13a-2-fkbp12::TRP1 fpr1::natMX6 tor1-1 rpb9-frb::hghMX*	This study
AKY2208	*MAT A, taf2-3xFlag::NAT fpr1::LEU2 rpl13a-2-fkbp12::TRP1 tor1-1 rpb9-frb::hghMX*	This study
AKY2209	*MAT A, taf14W81A::spHIS5 taf2-3xFlag::NAT fpr1::LEU2 rpl13a-2-fkbp12::TRP1 tor1-1 rpb9-frb::hghMX*	This study
AKY2268	*MAT A, taf14W81A::spHIS5 taf2Δ1261-1407-3xFlag::KanMX rpl13a-2-fkbp12::TRP1 fpr1::natMX6 tor1-1 rpb9-frb::hghMX*	This study
AKY719	*MAT α, rpb9::natMX6 bar1::hghMX*	This study
AKY1786	*MAT A, rpb4::spHIS5*	This study
AKY1818	*MAT α, taf14::KanMX*	This study
AKY1820	*MAT α, yaf9::TRP1*	This study
AKY1916	*MAT A, taf14::URA3*	This study
AKY1938	*MAT α, rpb4::LEU2*	This study
